# Oxidative Stress Risk Is Increased with a Sedentary Lifestyle during Aging in Mexican Women

**DOI:** 10.1155/2021/9971765

**Published:** 2021-10-25

**Authors:** Martha A. Sánchez-Rodríguez, Mariano Zacarías-Flores, Elsa Correa-Muñoz, Alicia Arronte-Rosales, Víctor Manuel Mendoza-Núñez

**Affiliations:** ^1^Research Unit on Gerontology, FES Zaragoza, National Autonomous University of Mexico, Mexico City, Mexico, Av. Guelatao No. 66, Col. Ejército de Oriente, Iztapalapa, Ciudad de Mexico, CP 09230, Mexico; ^2^Division of Obstetrics and Gynecology, Hospital Gustavo Baz Prada, Institute of Health of the State of Mexico, Nezahualcóyotl, State of Mexico CP 57300, Mexico

## Abstract

Oxidative stress (OS) increases during the human aging process, and the sedentary lifestyle could be a prooxidant factor. In this study, we determine the effect of sedentary lifestyle on OS during the aging process in Mexican women. A longitudinal study of two-year follow-up was carried out with 177 community-dwelling women (40-69 y) from Mexico City. We measured as OS markers plasma malondialdehyde, erythrocyte glutathione peroxidase (GPx) and superoxide dismutase (SOD), total plasma antioxidant status, uric acid level, antioxidant gap, and SOD/GPx ratio. To define OS using all the markers, we defined cut-off values of each parameter based on the 90^th^ percentile of young healthy subjects and, we calculated a stress score (SS) ranging from 0 to 7, which represented the intensity of the marker modifications. All the women answered a structured questionnaire about prooxidant factors, including physical activity specially the type of activity, frequency, and duration, and they answered Spanish versions of self-assessment tests for establishing dysthymia and insomnia as potential confounders. Principal component and Poisson regression analysis were used as statistical tools, being two-year OS the primary outcome. The OS was considerate as SS ≥ 4 and sedentary lifestyle as <30 min/day of physical activity, beside several prooxidant factors and age that were covariables. SS is higher in sedentary lifestyle women after the two-year follow-up; although, the difference was statistically significant only in older women. Four principal components were associated with the OS, and 7 out of 8 prooxidant factors were important for the analysis, which were included in the Poisson model. The predictive factors for OS were the sedentary lifestyle (adjusted PR = 2.37, CI_95%_: 1.30–4.30, *p* < 0.01), and age, in which the risk increases 1.06 (CI_95%_:1.02–2.11, *p* < 0.01) by each year of age. Our findings suggest that a sedentary lifestyle increases the OS during the aging in Mexican women.

## 1. Introduction

Since 1956, it was proposed that the aging is a biological process of every living organism which is related to oxidative stress (OS) [1], and this is defined as a biochemical imbalance between oxidants and antioxidant systems that produces an oxidative damage in biomolecules [2]. In humans, many investigations have been carried out to demonstrate an OS increment during the aging process [3, 4]; besides, there have been indicated several factors that accelerate or mitigate the relationship. Of these last factors, the intake of natural and artificial antioxidants, furthermore, the healthy lifestyle has been described as actions that promote healthy aging with less OS [5].

In women, the main changes produced by biological aging are associated with ovarian dysfunction during the menopausal transition with low production of estrogens, which causes many discomforting signs and symptoms such as hot flashes, night sweats, depression, headaches, insomnia, and lack of energy, among others [6, 7]. Most of these are prooxidant factors; thus, they favor the oxidation of biomolecules increasing the OS [8–10]. Likewise, as menopausal transition takes place, the women experience changes in their physical functioning which leads them to adopt to a more sedentary lifestyle [11]. Today, sedentary behavior is a characteristic of the population in general. This behavior has been recognized as a risk factor for a variety of diseases, mainly cardiovascular disease, and as a prooxidant risk factor [12].

Although this behavior is more prevalent in men than in women, the women acquire a sedentary lifestyle as the age progresses, starting around the fifth decade of life and is associated with the menopause [13, 14], as we indicated above. As other lifestyle factors, the sedentary lifestyle has been pointed out as a prooxidant factor because it has been related to the dysregulation of cellular redox status and diminished mitochondrial function [15]. Likewise, a study with muscle biopsies of sedentary aged humans has shown high intramuscular lipid peroxidation at rest and during acute exercise, finding the association between sedentary behavior, aging, and OS [16]. Although few studies confirm this relationship at the population level and much less in female aging, because the studies have focused mainly on men of different age groups or in older subjects, besides, these researchers have studied the antioxidant effect of physical activity. Thus, the aim of this study was to determine the effect of sedentary lifestyle on OS during the aging process in Mexican women.

## 2. Materials and Methods

### 2.1. Study Design and Participants

**Figure 1:**
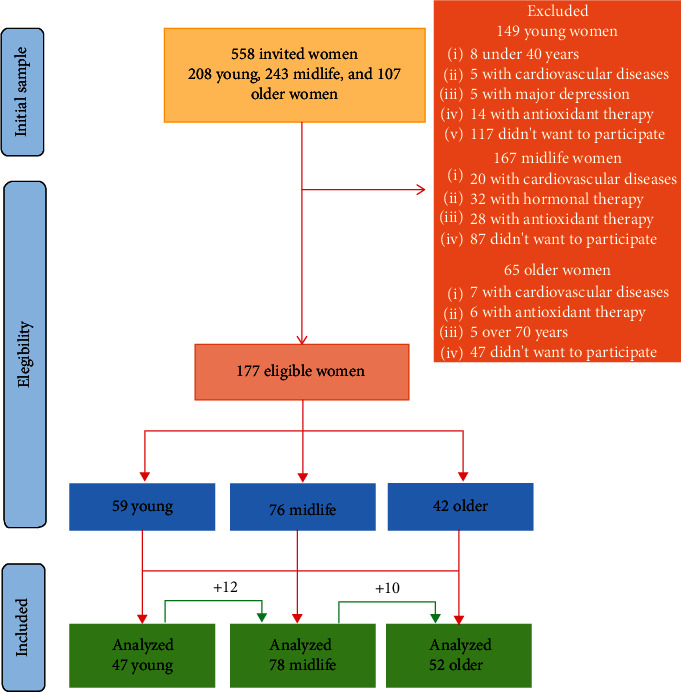
Flow diagram of the study.

An observational longitudinal study was carried out with 177 women (40-69 y) from Mexico City (healthy or with medical conditions under control namely hypertension and/or diabetes). The women were invited to participate in the Menopause and Oxidative Stress Project from the Research Unit on Gerontology at National Autonomous University of Mexico, Zaragoza Campus, from March to July 2017. The invitation was made through brochures specifying the eligibility criteria and objectives of the study. For this call, 558 women attended, and 383 potential participants were eliminated for different reasons such as the women with cardiovascular disease, hormonal or antioxidant therapy, or out of the age group, among others (Figure 1). The women with cardiovascular, kidney, hepatic, and cancer disease, assessed by medical history and physical examination, were eliminated. The women were separated into three groups according to decades of age: (i) 59 young women (40-49 y), (ii) 76 midlife women (50-59 y), and (iii) 42 older women (60-69 y). The participants had not previously taken any antioxidant supplement or hormone therapy, for at least six months prior to the beginning and throughout the study. The follow-up was for two years.

All participants agreed and signed an informed consent in order to participate in the study. This work is part of a clinical trial approved by the Ethics Committee of the National Autonomous University of Mexico (UNAM) Zaragoza Campus (register number FESZ/DEPI/CI/004/17) and recorded in the ISRCTN Registry Survey (ISRCTN14939779). In this study, follow-up of participants in the no intervention arm is shown.

To establish their health status, the participants were subjected to the following examinations: complete blood count, lipid profile and glucose, blood pressure, and anthropometric measurements, in addition to a complete clinical history. We used the cut-off points of reference values for Mexican population [17].

After 12-h fasting period, blood samples were collected by venipuncture. The samples were placed in vacutainer/siliconized test tubes with heparin as anticoagulant agent and a separating gel with no additives (Becton-Dickinson, Mexico City, Mexico). With the heparinized samples, we carried out the complete blood count in a Celly 70 auto analyzer (Chronolab, Mexico City, Mexico). The serum chemical measurements of glucose, total cholesterol, triglycerides, and high-density lipoprotein cholesterol (HDL-c) levels were tested using a Cobas C111 analyzer (Roche Diagnostics, Basilea, SW). As quality control, the intra- and interassay variation coefficients were obtained, which were less than 5% in all determinations.

The weight of each woman was measured in a fasting state (after evacuation), and they wore a clinic gown and underwear using a Torino® scale (Tecno Lógica, Mexicana, Mexico, TLM®) calibrated before each weight measurement. The height was obtained with an aluminum cursor stadiometer (graduated in millimeters). For this, the woman was placed on the stadiometer in the Frankfurt horizontal plane, and she stood barefoot, backward, and with the head in contact with it. We calculate body mass index (BMI) by dividing weight (in kilograms) through squared height (in meters). In accordance with international guidelines, the BMI cut-off value was equal or greater than 25.0 kg/m^2^ [18]. A mercurial manometer was used to measure each woman blood pressure in both arms in the morning, in sitting position after resting for 5 minutes and in a fasting condition; the mean of these measurements was reported as result. All the measurements were carried out by medical technicians trained in previous sessions for standardization of the procedures (intertechnician kappa coefficient = 0.95, *p* < 0.0001), and they were monitored to avoid biases in the measurements.

### 2.2. Sample Size

The sample size was calculated based on a one mean known principle detect a difference of 0.25 stress score (SS) points like the results found in a 6-month interventional study of low impact exercise vs. nonintervention [19]. To compute the sample size with the tables for clinical studies [20], a power of 80% and a 5% level of significance were used because it is a longitudinal study, and it was added 20% more to compensate possible lost during the follow-up. The final size was 154 women, but we selected 177 women who meet the eligibility criteria to ensure the results.

### 2.3. Oxidative Stress Measurement

With the heparinized samples, we measured plasma malondialdehyde level (MDA), red blood cell glutathione peroxidase (GPx), superoxide dismutase (SOD) activities, and plasma total antioxidant status (TAS). In our research laboratory, the methods were validated (within run precision values: 6%, 4.6%, 3.8%, and 4.3%, respectively). We added 10 *μ*L of 2 mM butylated hydroxytoluene in ethanol at 95% immediately after centrifugation, and artificial formation of thiobarbituric acid reacting substances (TBARS) in the samples was prevented.

The MDA level was measured using the TBARS assay described by Jentzsch et al. [21]. This procedure was previously validated by our research group. To avoid the amplification of peroxidation during the assay, the chain-breaking antioxidant butyryl hydroxytoluene (BHT) (Sigma Chemical Co. St. Louis, MO, USA) was added to the heparinized sample. Briefly, in this procedure, 400 *μ*L of sample or 1,1,3,3-tetramethoxypropane (TMP) (Sigma Chemical Co.), used as MDA standard (0.2–4 mmol/L), was mixed with 400 *μ*L orthophosphoric acid (0.2 mol/L, Sigma Chemical Co.) and 50 *μ*L BHT (2 mmol/L). After 50 *μ*L of thiobarbituric acid (TBA) (0.11 mol/L in 0.1 mol/L NaOH; Fluka Chem., Buchs, Switzerland) reagent was added, the mix was placed in a water bath at 90°C for 45 min. After cooled in ice to stop the reaction, TBARS were extracted with 1000 *μ*L *n*-butanol (Sigma Chemical Co.). The butanol phase was read at 535 nm and 572 nm to correct baseline absorption. The absorption difference was used to calculate MDA concentration in the samples using the calibration curve.

GPx, SOD activities, and total antioxidant status (TAS) were performed using commercial kits according to the manufacturer's instructions (Randox Laboratories, Ltd.). In GPx test, cumene hydroperoxide in the presence of glutathione reductase and NADPH produces the oxidation of glutathione, which is immediately converted into the reduced form with the subsequent oxidation of NADPH to NADP+, which is measured at 340 nm. In the method to determinate SOD activity, superoxide radicals were generated using xanthine and xanthine oxidase; the superoxide radicals react with 2-(4-iodophenyl)-3-(4-nitrophenol)-5-phenyltetrazolium chloride to form a red formazan dye. The TAS test determines the decrease in absorbance that is proportional to the blood antioxidant concentration of the sample, and the method measures 2,2-azino-bis (3-ethylbenzthiazoline-6-sulfonic acid, ABTS^+^) radical formation kinetics. All the measurements were performed in a Shimadzu UV-1601 UV-Vis spectrophotometer (Kyoto, Japan). Also, uric acid by the uricase colorimetric method and albumin levels using bromocresol green technique, with a Cobas C111 analyzer, were assessed. The intrarun precision assays were 2.8% and 3.1%, respectively.

Additionally, we obtained the SOD/GPx ratio and the antioxidant gap (GAP) with the follow equation:

GAP = TAS − [(albumin (*μ*mol) × 0.69) + uric acid (*μ*mol)] [22].

To define OS using all the markers, we defined cut-off values of each parameter based on the 90^th^ percentile of young healthy subjects. The cut-off values were MDA ≥ 0.320 *μ*mol/L, GPx ≤ 50.1 U/gHb, SOD ≤ 1.20 U/gHb, TAS ≤ 1030 *μ*mol/L, SOD/GPx ≥ 0.023, and GAP ≤ 278 *μ*mol/L. The cut-off values of the uric acid were the median of the reference interval (>268 *μ*mol/L for adult woman and 315 *μ*mol/L for an older woman) as determined to the Mexican population [17]. A score of 1 was given to each value higher or lower than the cut-off point established, and then we sum all the parameters, computing an oxidative stress score (SS) ranging from 0 to 7, which represented the intensity of the marker modifications. A cut-off value of ≥4 was considered as OS, which is the median value of SS as previously was proposed [23].

### 2.4. Evaluation of Dysthymia, Insomnia, and Prooxidant Lifestyle Factors

The women answered Spanish versions of self-assessment tests for established the possibility of dysthymia and insomnia as potential confounders and a structured questionnaire about prooxidant factors to know about their physical activity and other lifestyle habits.

For dysthymia, the young and midlife women answered the Zung Self-Rating Depression Scale (SDS), whose cut-off value to considerate as normal is an SDS score below 40 [24, 25]. The older women completed the 30-item Geriatric Depression Scale (GDS) in which a woman with a score ≤ 10 had not depression [26]. Major depression was diagnosed according to Diagnostic and Statistical Manual of Mental Disorders (DSM-5). Because both scales are different, the results were transformed into a normalized score, obtaining a percentage index multiplying the points obtained from each woman by 100 and dividing by 80 for SDS or 30 for GDS.

To evaluate sleep disturbances, we used the Athens Insomnia Scale (AIS), a validated psychometric instrument based on the ICD-10 criteria, designed to determine sleep difficulty. It is a self-assessment, in which a cut-off value of ≥8 was considered as insomnia [27, 28].

Also, the women completed a structured questionnaire assessing the habits: smoking, intake of alcoholic and/or caffeinated beverages, and physical activity pointed type of activity, frequency, and duration, as lifestyle prooxidant factors. A prooxidant factor present was considerate if smoking ≥ 2 cigarettes/day, intake of ≥2 glasses/day alcoholic beverages (beer or spirits), intake of >2 cups/day highly caffeinated beverages, and <30 min/day of physical activity (sedentary lifestyle).

### 2.5. Follow-Up Phase

All the measurements were carried out every 6 months for up to two years, to keep the sample. One person of our research group was in contact by phone with the participants each month during the follow-up time, ensuring that no women dropped out of the study, and they did not take hormone therapy or antioxidant supplements.

### 2.6. Statistical Analysis

Quantitative data were presented as the mean and standard deviation and categorical data as frequency, percentage, and 95% confidence interval (CI_95%_). We used one-way ANOVA with Tukey's posthoc test and Pearson's chi-squared test, respectively, to compare them. We corroborate normal distribution of continuous data with the Kolgomorov-Smirnoff test and homogeneity of variances with the Levene test. To evaluate the proportions before and after two years, McNemar chi-squared test was used, and to compare the means, paired t-test was calculated. A repeated measures multivariate analysis of variance was conducted to investigate the change of oxidative stress parameters over time. The between-subject factor was each age group, the within-subject factor was time (baseline versus 2 years), and Tukey's test was calculated as posthoc. Also, two factors repeated measures analysis of variance was carried out, adding to the model the dichotomous sedentary lifestyle as an another between-subject factor.

The multivariate technique of Principal Component Analysis (PCA) was used to systematically reduce the number of dimensions needed to describe OS. The prooxidant factors age, BMI, AIS score, dysthymia normalized score, number of cigarettes, cup of caffeine beverages, glasses of alcohol, and duration of physical activity were used to perform the PCA. Barlett's test of sphericity and the measure of sampling adequacy score of Kaiser-Meyer-Olkin (KMO) were obtained, to assess the homogeneity of variances among the different eigenvalues correlated to the principal component, and in which degree each variable may be predicted by all the other variables, respectively. A KMO value higher than 0.5 indicates that the solution obtained with PCA can be accepted. Because the data are not dimensionally homogeneous, the PCA was calculated with the method based on the correlation matrix, in which only the PCs eigenvalue greater than 1 was considerate, to assure that the set of PCs are more important than the original variables. In addition, an orthogonal rotation was performed by the varimax method to maximize the variance of the factors. When the correlation result of a factor was higher than 0.5, it was considered highly correlated with a PC. A three-dimensional plot was constructed in which the variables are positively correlated when they form an angle of 0 degrees from the origin and negatively when the angle is 180 degrees. An angle of 90 degrees indicates that the variables are uncorrelated [29, 30].

To assess the risk of each factor selected by the PCA, the Poisson regression test was used for multivariate analysis being as primary outcome the OS after 2 years, considering the cut-off value of SS ≥ 4. The models were adjusted by the dichotomous variables smoking, intake of alcoholic beverages, intake of caffeinated beverages, overweight/obesity, dysthymia, insomnia, and sedentary lifestyle, as factors and age at baseline as quantitative covariable. The prevalence ratio (PR) and CI_95%_ were obtained. A risk factor was considerate if PR > 1 and the 95% CI did not include the 1.0 values. A two-tailed *p* value <0.05 was considerate as significant. The standard statistical software package SPSS V. 20.0 (IBM SPSS Statistics Armonk, NY, USA) was used for the processing of the data.

## 3. Results

### 3.1. Sample Characteristics

We observed that all the women completed the follow-up, and after 2 years, 12 (20%) young women passed to the midlife age group, and 10 (13%) midlife women passed to the older women group (Figure 1).

At basal time, young and midlife groups were similar in all the parameters, but the older women had higher glucose levels, and the proportion of diabetes mellitus and arterial hypertension is more frequent than the other groups. Regardless of the lifestyle factors, women who smoke or drink alcohol are few, with a prevalence ranging between 9 and 15% (Table 1).

The physical activity was different between the age group, the older and the midlife women frequently walk ≥ 30 min/d, and the young women running ≥ 30 min/d. After two years, 95 (54%) women still with physical activity, and 6 (12%) old women became sedentary, but the change was not significant (Table 2).

### 3.2. Oxidative Stress by the Age Group

About oxidative stress markers, antioxidant enzymes were significantly lower in older women, and the SOD/GPx ratio and uric acid levels were higher, compared with young and midlife women. The malondialdehyde level did not change after two years. SOD activity, total antioxidant status, and antioxidant gap change over the time and shown an age group time interaction. The stress score is higher in older women than the other groups, and it increases after the two-year period in these women (Table 3).

### 3.3. Oxidative Stress and Sedentary Lifestyle

**Figure 2:**
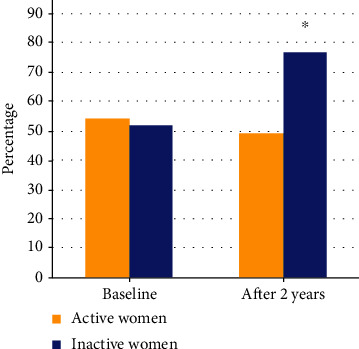
Percentage of women with oxidative stress at baseline and after two-year follow-up, separated by the active lifestyle. McNemar chi-squared test, ^∗^*p* < 0.001.

**Figure 3:**
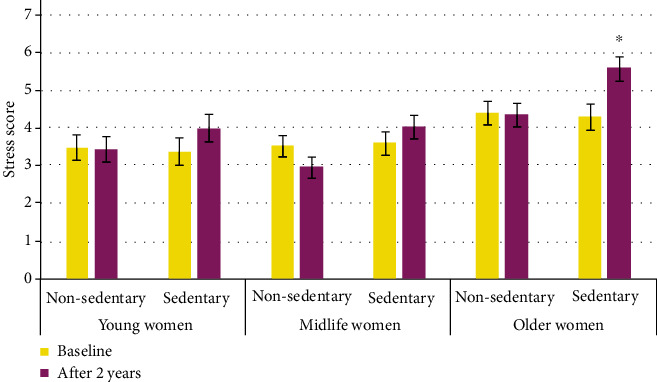
Stress scores by age groups at baseline and after two-year follow-up, separated by the sedentary lifestyle. Data shows mean and standard error. Two-way repeated measures ANOVA with paired *t*-test as posthoc, ^∗^*p* < 0.05.

Concerning OS and sedentary lifestyle association, at baseline, 40 (52%) of inactive women had high OS and after the two-year period, the proportion increased to 59 (77%), *p* < 0.001 (Figure 2). Likewise, stress score is higher in sedentary lifestyle women after the two-year follow-up; although, the difference was statistically significant only in older women; the OS score did not change in all groups of nonsedentary women (Figure 3).

To assess which prooxidant factors are most associated with OS, a PCA was performed. Preliminary analysis to verify the adequacy of the data for a factorial analysis showed a significant value of Barlett's test of sphericity (*p* < 0.0001) that allows formally contrasting the existence of correlation between the variables, and KMO = 0.514, which indicate that the PCA can be accepted. Four PC were selected, corresponding to 67.7% of the total variance, in which 7 out of 8 prooxidant factors distributed in the four PC and were important for the analysis. In Table 4, the main factors for OS are presented in order of importance, in which the correlation value approaching “-1 or 1” indicates that the variable significantly affects the component. BMI had low correlation; thus, it was discarded. Principal component 1 (PC1) included the dysthymia normalized score and age as higher correlation factors and accounted for 23% of the overall variance. The other three components accounted between 13% and 17% of overall variance and included the other five prooxidant factors. The dysthymia normalized score and glasses of alcohol have a negative correlation.

**Figure 4:**
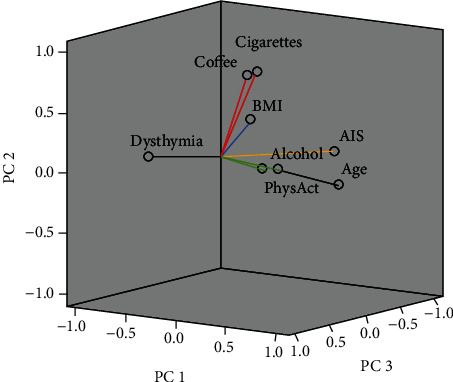
Three-dimensional plot of principal component analysis in rotate space notices how the variables age, AIS scores, glasses of alcohol, and minutes of physical activity form a cluster with small angles between each, and the dysthymia index is in the opposite side. A second group is formed by number of cigarettes and cups of caffeinated beverages. The variables with black lines are in PC1, with an orange line that is in PC3, with green lines that are in PC4, and with red lines that are in PC2. BMI had low correlation (blue line). AIS: Athens Insomnia Scale; alcohol: glasses of alcohol; BMI: body mass index; cigarettes: number of cigarettes; coffee: cups of caffeinated beverages; dysthymia: dysthymia index; PC: principal component; PhysAct: minutes of physical activity.

In addition, the graphical representation of the component weights after rotation is shown in Figure 4. This plot shows the first three PCs which explains ~55% of the total variance. As is difficult to see in a three-dimensional plot, the PC in which each variable is found, and a colored line was placed to indicate the PC to which it belongs. It is observed that the prooxidant factors: age (PC1), AIS scores (PC3), glasses of alcohol (PC4), and minutes of physical activity (PC4) form a cluster with small angles between each, and the dysthymia index (PC1) is on the opposite side. A second group is formed by number of cigarettes and cups of caffeinated beverages, and they are located in the PC2.

Because the PCA only indicates a cluster of prooxidant factors correlated with OS, but it is not easy to determinate the weight of each one, a Poisson regression was performed. In the risk analysis, the predictive factors for OS were the sedentary lifestyle (adjusted PR = 2.37, CI_95%:_1.30–4.30, *p* < 0.01) and age, in which by each year of age that increases, the risk is 1.06 (CI_95%_:1.02–2.11, *p* < 0.01), and other prooxidant factors did not risk for the OS (Table 5).

## 4. Discussion

Aging is a complex process that involves the time and the biological and environmental factors with the outcome of an imbalance between damage and repair of biomolecules, in which damage wins because the repair pathways gradually decline over the time, having the OS an important role as it participates in these reactions [31, 32]. Being this a complex process, there is no direct relationship between OS and aging; so, there are external factors (prooxidants), such as lifestyle factors, that accelerate it due to a possible decrease in antioxidants and alteration of the immune system [33]. The role of sedentary lifestyle in aging OS relationship has been studied mainly in basic research [16, 34, 35]; therefore, it was our interest to assess the effect of this risk factor in the causal chain OS aging.

Most of the evidence shows that the physical activity changes across the life tending to a decrease during the aging process; however, also, an increment in leisure time physical activity is reported after the retirement transition, because the people have more time to do exercise [36], causing a controversial information. In women, it is recognized that after menopause, the physical functioning status decreases over the time, probably associated with physiological and symptomatic changes of this stage [11], causing a more sedentary lifestyle. Within this controversial information, the older women included in this study had more physical activity than the younger and middle-aged women; although, the exercise they perform is less impact since it is walking, compared with running or swimming. This is possible because, as we pointed out before, older women are concerned about their health and take on fewer social roles, different from younger and midlife people who are in a productive stage with high participation in the community [37]; thus, they have less time to dedicate for their health. Likewise, the benefit of the practice of moderate exercise is known to all. In this sense, exercise causes a hormetic response due an adaptive process to exposure to low quantities of stressors such as moderate physical activity, producing several beneficial effects like an improvement on cardiovascular function, and upregulates the immune system, delayed aging, and modulation of redox homeostasis with an increment of antioxidant response, among others [38, 39]; thus, the sedentary lifestyle has the opposite effect. Also, a review that analyzes the effect of prolonged sitting time on cardiometabolic risk recommended changing this behavior to a more active one to counteract the detrimental effect of sedentary lifestyle, according to the subjects' characteristics [40].

As we pointed above, the relationship between aging and OS had been described in several studies using different OS markers. In this study, we calculated an integrated stress score that involves both oxidized molecules and antioxidants, which corroborate that the OS is higher in older women, and after two years of follow-up, the OS only increases in this age group, corroborating the relationship, as previously it was reported in cross-sectional studies using individual markers [3, 41]; although, a research has recently noted that only some OS markers change in adults over 55 years [42]. In this sense, in our study, lowest antioxidant components were observed in older women, but this is not necessarily indicative of the OS; therefore, it seems to point the importance to integrate the markers to define when a subject has an OS [43].

Several lifestyle factors are considered prooxidants because increase the OS. The PCA was performed to reduce the number of variables, because linear combination of the original variables is readily interpretable, therefore, each PC has a linear combination of the original variables [29]. This analysis revealed four PC containing 7 prooxidant factors. The PC1 explains the 23% of the variance, in which the age emerges as the main prooxidant factor positively associated with the OS and negatively related to dysthymia. Concerning this, the OS increases with the age [3], and the dysthymia is more frequent in middle-aged women because they may be going through the menopausal transition, causing OS [9], that is why they are opposites in the same component. Secondly, AIS scores (PC3), glasses of alcohol (PC4), and minutes of physical activity (PC4) are close, as the three-dimensional plot shows, indicating that these factors are interrelated, and they are not easy to separate to assess their effect. In addition, cups of caffeinated beverages and the number of cigarettes are in a second cluster group, and they are also variables to consider. The role of all those factors as prooxidants had been shown individually; although, the role of some remains unclear, and the association in a multivariate analysis is not common [44–47].

In PCA, the minutes of physical activity were introduced to the model, considering that our interest was to measure sedentary as a lifestyle with serious consequences during the aging. As well as the other factors, they are in a quantitative scale; thus, we transform all the variables in dichotomous scale to specify the risk to OS trying to separate the effect. In the risk analysis, only sedentary lifestyle and the age were risk factors for OS, and the other lifestyle prooxidant behaviors were not related. These results are consistent with the report of a study of factors associated with healthy aging of the Mexican population; the authors point out that smoking, alcohol intake, depression, and overweight/obesity are not related to the dependent variable [48]. Although a different association was sought, it seems that these variables are not related to events in which the age is included in the models. It is important to notice that age is the main risk factor for OS, independently if the analysis is with quantitative or categorical data, topic extensively discussed in the literature, but the aim of our study is to try to clarify the effect of the other pro-oxidant factors.

Likewise, our results have shown that the sedentary lifestyle increases the OS after a two-year period, mainly in older women, and it is a risk factor for OS. Thus, the women with sedentary lifestyle had twofold risk for OS, plus 6% by each year of age; therefore, sedentary lifestyle seems to accelerate the OS during the aging, advancing the probability of tissue oxidative damage and incrementing the probability to develop the ageing-related diseases due to the OS, as previously has been indicated [49]. As for, it has been described that sedentary behavior is associated with mitochondrial oxidative damage in muscle [34] and enhances vascular reactive oxygen species production, which contributes to endothelial dysfunction and atherosclerosis [35]; hence, a sedentary lifestyle is associated with cardiovascular risk and loss of skeletal muscle mass and ageing-related events. Under such circumstances, it is recognized that a sedentary lifestyle is associated with depression, all-cause mortality, and cause of frailty in old people [50–52], events related to OS.

Furthermore, some studies showed that OS increases in elderly, although not significant, after 6 months of follow-up without intervention [53]. Therefore, the fact that with each year of age, the risk of OS increases besides sedentary lifestyle, and it is an additional contribution to studies of the harmful effect of this behavior and supports the research that has shown the benefit of low-impact exercise on OS and other health parameters [19, 54]. In this context, it is important to highlight that in Mexico more than 65% of the population aged 50 and over has a sedentary lifestyle [54].

The limitations of the study include the small sample size and the study follow-up time. Although it is important to highlight as strengths of the research the longitudinal design and the low drop of the participants of the cohort, in addition to the proposal to analyze the role of sedentary lifestyle instead of exercise. However, more longitudinal studies are needed to verify our findings.

## 5. Conclusions

It is important to note that in the two-year follow-up cohort of Mexican women in the aging process, the older subgroup (60 to 69 years) had significantly higher systolic blood pressure compared to the groups of young women (40 to 49 years) and middle-aged (50 to 59 years) (*p* < 0.001), in addition to a higher prevalence of diabetes mellitus in the older subgroup. Also, this group (60 to 69 years) was the one that reported a significantly higher percentage regard to continuing with physical activity (*p* < 0.001). Likewise, a significantly higher oxidative stress index was found in the subgroup of older women compared to the younger and middle-aged subgroups (*p* < 0.05). On the other hand, in the global analysis of the population, a positive correlation was observed between age, consumption of coffee cups, number of cigarettes, and score on the insomnia scale with the oxidative stress index, in contrast to a negative association with the moderate consumption of alcoholic beverages and the score on the scale to assess dysthymia. Finally, the most relevant finding of our study was that regardless of age and other prooxidant factors, sedentary lifestyle constitutes a statistically significant risk factor for oxidative stress Mexican women in the aging process.

These findings support the proposal to implement programs that strengthen healthy lifestyles during early aging.

## Figures and Tables

**Table 1:** Baseline characteristics of study groups.

Parameter	Young women (*n* = 59)	Midlife women (*n* = 76)	Older women (*n* = 42)
Age (years)	46 ± 2	54 ± 3^a^	64 ± 3^b^
Hemoglobin (g/dL)	14.1 ± 1.4	14.4 ± 1.1	14.6 ± 0.9
Glucose (*μ*mol/L)	5.3 ± 2.0	5.9 ± 2.5	6.7 ± 3.2^c^
Cholesterol (*μ*mol/L)	5.6 ± 1.0	5.6 ± 1.2	5.8 ± 1.3
Triglycerides (*μ*mol/L)	2.1 ± 1.1	2.1 ± 1.4	2.5 ± 2.4
HDLc (*μ*mol/L)	1.5 ± 0.4	1.4 ± 0.4	1.4 ± 0.4
Body mass index (kg/m^2^)	28.82 ± 4.4	28.88 ± 5.0	27.89 ± 4.8
Systolic blood pressure (mm hg)	117 ± 13	123 ± 15	135 ± 28^b,d^
Diastolic blood pressure (mm hg)	79 ± 8	81 ± 8	82 ± 14
Smoking (≥ 2 cigarettes/d)	8 (14%, 6–22%)	7 (9%, 3–15%)	5 (12%, 2–22%)
Highly caffeine beverage intake (≥ 2 cups/d)	20 (34%, 22–46%)	31 (41%, 30–52%)	22 (52%, 37–67%)
Alcohol beverages intake (≥ 2 cups/d)	6 (10%, 2–18%)	8 (11%, 3–18%)	6 (15%, 4–26%)
Sedentary lifestyle (< 30 min of physical activity/d)	27 (46%, 33–59%)	36 (47%, 36–58%)	14 (33%, 19–47%)
Insomnia (Athens insomnia scale >8)	23 (39%, 27–51%)	44 (56%, 45–67%)	16 (38%, 23–53%)
Dysthymia	14 (24%, 13–35%)	24 (32%, 22–42%)	13 (31%, 17–45%)
Diabetes mellitus	4 (7%, 1–14%)	11 (15%, 7–23%)	12 (29%, 15–43%)^e^
Arterial hypertension	16 (27%, 16–38%)	32 (42%, 31–53%)	26 (62%, 47–77%)^e,f^

Continuous data show means ± standard deviation; categorical data show frequency, percentage. and 95% confidence interval. One-way ANOVA test with Tukey test as posthoc. ^a^Young vs. midlife, *p* < 0.0001; ^b^young vs. older, *p* < 0.0001; ^c^young vs. older, *p* < 0.05; ^d^midlife vs. older, *p* < 0.01. *χ*^2^ test, ^e^young vs. older, *p* < 0.0001; ^f^midlife vs. older, *p* < 0.05.

**Table 2:** Physical activity performed at the beginning and after two years by study groups.

Physical activity	Young women	Midlife women	Older women
Walking (≥ 30 min/d)			
Baseline	4/59 (7%, 1–14%)	19/76 (25%, 15–35%)^a^	19/42 (45%, 30–60%)^b,c^
After two years	1/47 (2%, 0–6%)	16/78 (21%, 12–30%)	20/52 (39%, 26–52%)^d^
Aerobics (60 min/d)			
Baseline	7/59 (12%, 4–20%)	12/76 (16%, 8–24%)	3/42 (7%, 3–11%)
After two years	6/47 (13%, 3–23%)	8/78 (10%, 3–17%)	7/52 (13%, 4–22%)
Yoga (60 min/d)			
Baseline	4/59 (7%, 0–14%)	4/76 (5%, 0–8%)	6/42 (14%, 4–24%)
After two years	3/47 (6%, 0–13%)	7/78 (9%, 3–15%)	5/52 (9%, 1–17%)
Running (≥ 30 min/d)			
Baseline	13/59 (22%, 11–33%)	5/76 (7%, 1–13%)^e^	0
After two years	12/47 (26%, 10–20%)	6/78 (8%, 2–14%)^f^	0
Swimming (60 min/d)			
Baseline	4/59 (7%, 1–14%)	0	0
After two years	3/47 (6%, 0–13%)	1/78 (1%,0–2%)	0
Without physical activity			
Baseline	27/59 (46%, 33–59%)	36/76 (47%, 36–58%)	14/42 (33%, 19–47%)^g,h^
After two years	22/47 (47%, 33–61%)	40/78 (51%, 40–62%)	20/52 (39%, 27–53%)

Data show frequency, percentage, and 95% confidence interval. *χ*^2^ test, ^a^young vs. midlife, *p* < 0.01; ^b^young vs. older, *p* < 0.0001; ^c^midlife vs. older, *p* < 0.05; ^d^young vs. older, *p* < 0.0001;^e^young vs. midlife, *p* < 0.0001; ^f^young vs. midlife, *p* < 0.01;^g^young vs. older, *p* < 0.01; ^h^midlife vs. older, *p* < 0.001.

**Table 3:** Baseline and after two years oxidative stress markers by study groups. Data show mean and standard deviation.

Oxidative stress marker	Baseline			After two years		
	Young women (*n* = 59)	Midlife women (*n* = 76)	Older women (*n* = 42)	Young women (*n* = 47)	Midlife women (*n* = 78)	Older women (*n* = 52)
Malondialdehyde (*μ*mol/L)	0.323 ± 0.07	0.336 ± 0.07	0.329 ± 0.12	0.328 ± 0.06	0.338 ± 0.06	0.341 ± 0.09
Superoxide dismutase (U/g Hb)^∗^^†^	1.22 ± 0.1	1.19 ± 0.1	1.12 ± 0.1	1.27 ± 0.2	1.21 ± 0.1^a^	1.19 ± 0.1^b^
Glutathione peroxidase (U/g Hb)	55.5 ± 15.9	53.3 ± 16.5	41.9 ± 17.5	55.7 ± 16.5	53.6 ± 14.4	46.9 ± 19.0^c,d^
Uric acid (*μ*mol/L)	264 ± 68	275 ± 76	309 ± 82^d^	255 ± 68	297 ± 74	313 ± 91^e^
Total antioxidant status (*μ*mol/L)^‡§^	1018 ± 226	1088 ± 251	1071 ± 226	1015 ± 236	1105 ± 232	913 ± 188^f^
Antioxidant gap (*μ*mol/L)^‡§^	287 ± 232	338 ± 239	312 ± 237	311 ± 247	347 ± 221	156 ± 251^f^
SOD/GPx ratio	0.024 ± 0.007	0.025 ± 0.008	0.031 ± 0.012	0.024 ± 0.007	0.024 ± 0.007^d^	0.029 ± 0.010^g^
Stress score	3.42 ± 1.85	3.55 ± 1.82	4.36 ± 1.51	3.69 ± 1.65	3.46 ± 1.77	4.76 ± 1.62^e,g^

Repeated measures analysis of variance. ^∗^Change over the time, *p* < 0.0001; ^†^age group-time interaction, *p* < 0.05; ^‡^change over the time, *p* < 0.05; ^§^age group-time interaction, *p*<0.01. Tukey's test as posthoc. ^a^Young vs. midlife, *p* < 0.05; ^b^young vs. older, *p* < 0.001; ^c^young vs. older, *p* < 0.0001; ^d^midlife vs. older, *p* < 0.001; ^e^young vs. older, *p* < 0.01; ^f^midlife vs. older, *p* < 0.05; ^g^midlife vs. older, *p* < 0.0001.

**Table 4:** Main factors to oxidative stress obtained by component principal analysis with orthogonal rotation.

Principal component	Prooxidant factor	Correlation *r*	Percentage of variance %
1	Age Dysthymia normalized score	0.854 -0.842	23.2
2	Cups of highly caffeinate beverage Number of cigarettes	0.791 0.756	17.0
3	Athens insomnia scale score	0.881	14.3
4	Duration of physical activity Glasses of alcohol	0.812 -0.543	13.1

**Table 5:** Predictors of the outcome oxidative stress in 40 to 69 years women after a two-year period.

Predictor	Prevalence ratio	CI_95%_	*p* value^∗^
Sedentary lifestyle (yes)	2.37	1.30–4.30	0.005
Age (years)	1.06	1.02–1.11	0.003
Smoking (yes)	1.46	0.56–3.81	0.438
Alcohol beverages intake (≥ 2 glasses/d)	1.20	0.38–3.76	0.751
Dysthymia (yes)	1.13	0.58–2.20	0.725
Highly caffeine beverages intake (≥ 2 cups/d)	0.94	0.52–1.71	0.846
Insomnia (yes)	0.79	0.43–1.46	0.455

^∗^Poisson regression, model *p* value <0.01; CI_95**%**_: 95% confidence interval.

## Data Availability

The data used to support the findings of this study are available from the corresponding author upon request
